# Shape and Compliance of Endothelial Cells after Shear Stress In Vitro or from Different Aortic Regions: Scanning Ion Conductance Microscopy Study

**DOI:** 10.1371/journal.pone.0031228

**Published:** 2012-02-16

**Authors:** Claire M. F. Potter, Sophie Schobesberger, Martina H. Lundberg, Peter D. Weinberg, Jane A. Mitchell, Julia Gorelik

**Affiliations:** 1 Cardiothoracic Pharmacology, Pharmacology and Toxicology and Functional Microscopy, Cardiovascular Sciences, National Heart and Lung Institute, London, United Kingdom; 2 Department of Bioengineering, Imperial College London, London, United Kingdom; Idaho State University, United States of America

## Abstract

**Objective:**

To measure the elongation and compliance of endothelial cells subjected to different patterns of shear stress in vitro, and to compare these parameters with the elongation and compliance of endothelial cells from different regions of the intact aorta.

**Materials and Methods:**

Porcine aortic endothelial cells were cultured for 6 days under static conditions or on an orbital shaker. The shaker generated a wave of medium, inducing pulsatile shear stress with a preferred orientation at the edge of the well or steadier shear stress with changing orientation at its centre. The topography and compliance of these cells and cells from the inner and outer curvature of ex vivo porcine aortic arches were measured by scanning ion conductance microscopy (SICM).

**Results:**

Cells cultured under oriented shear stress were more elongated and less compliant than cells grown under static conditions or under shear stress with no preferred orientation. Cells from the outer curvature of the aorta were more elongated and less compliant than cells from the inner curvature.

**Conclusion:**

The elongation and compliance of cultured endothelial cells vary according to the pattern of applied shear stress, and are inversely correlated. A similar inverse correlation occurs in the aortic arch, with variation between regions thought to experience different haemodynamic stresses.

## Introduction

It has long been known that endothelial cells are rounded and randomly oriented when cultured under static conditions, but become elongated and aligned with the flow when exposed to a unidirectional shear stress [1]. Non-reversing pulsatile shear has a greater elongatory effect than steady shear of the same mean magnitude, reversing pulsatile shear has a lesser effect and oscillatory shear has no effect at all [2]. Endothelial cell border and nuclear elongation have been used to assess local shear stresses occurring in vivo and hence to explore the relation between shear and susceptibility to atherosclerosis [3], [4]. It has been proposed that more rounded cells, found in areas prone to plaque formation and predicted to be exposed to low shear stress, are dysfunctional and thus much has been done to investigate the physical properties of cells from both susceptible and protected regions of the vasculature and identify the differences between them. Initial studies focused on assessment of cell nuclear elongation and alignment in vivo [5] whilst later work went on to focus on cell morphology both in vivo [6], [7] and in vitro [1], [8], [9]. An increasing body of work suggests that cell morphology change is driven by cytoskeletal rearrangement [10], [11], [12] though the full mechanisms behind the mechanotransduction of shear stress remain unclear.

One area of focus in the evaluation of cell structure is measurement of local cell mechanobiology. It is known that on exposure to shear stress endothelial cell movement is reduced whilst polymerisation of filamentous actin is increased and actin stress fibres form in alignment with the direction of flow. These stress fibres are thought to cause a fall in the compliance of the endothelial cell membrane [13]. Endothelial cell membrane compliance has been measured by a number of methods. Such methods include those exerting a pulling force such as use of optical tweezers, whereby a bead is trapped in position over a cell and manipulated to make contact with the cell and then repositioned pulling on the cell membrane, magnetic rheology, in which an integrin coated bead is bound to a cell and manipulated by magnets [14] and micropippete aspiration [13] whereby suction pressure is used to pull the cell surface up into a glass tube. Though these methods allow for application of a range of forces they are limited in their ability to study cells in their native context, often requiring cells to be in suspension and require direct and potentially damaging contact with the cell. Other methods such as atomic force microscopy where the cell is probed with a cantilever exert a force pushing into the cell [15] but again this level of contact could be potentially damaging giving rise to artefacts potentially influencing results. The majority of the above methods are also limited in their spatial or temporal abilities and can only record one feature at a time. With the development of Scanning Ion Conductance Microscopy (SICM), such restrictions can be avoided. By allowing scanning and mechanical testing with minimal invasiveness, SICM is ideal for the investigation of living cells, enabling accurate recording of cell properties in their native context and when grown in vitro [16], [17].

We recently characterised a model to subject endothelial cells to different patterns of shear stress [18]. By placing circular culture wells on an orbital shaker, cells at the edge of the well are exposed to shear that fluctuates in magnitude and has a preferred (tangential) orientation whilst cells in the centre of the well are exposed to shear of a uniform magnitude (with the same mean value as at the edge) that fluctuates between radial and tangential orientations. We showed that cells at the edge are more aligned than those at the centre [18]. Here we use this model in conjunction with SICM to investigate whether porcine aortic endothelial cells (PAEC) differ in elongation and compliance depending upon the shear profile they experience. We additionally investigated these properties in the inner and outer curvature of the ascending porcine aorta.

## Materials and Methods

### Isolation and culture of porcine aortic endothelial cells (PAEC)

Cells were isolated from the descending thoracic aortas of Landrace cross pigs (Fresh Tissue Supplies, East Sussex, UK) by collagenase digestion as described in the method of Bogle et al [19]. These aortas were collected in Hank's Balance Salt Solution (HBSS) supplemented with antibiotics (All Sigma UK), delivered within 24 hours of animal slaughter and stored at 4°C until use. All cells used in experiments were at passage 2.

### Dissection of porcine aortas

**Figure 1:**
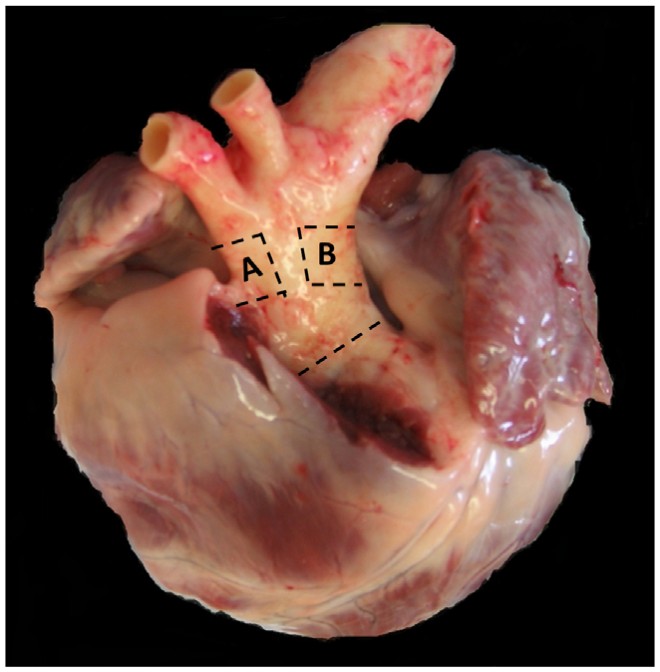
Image of a porcine heart and aorta illustrating the regions examined in this study. A is a section of the outer curvature, and B the inner curvature, of the ascending proximal aorta.

Porcine hearts with attached aortas were obtained directly from the abattoir (Cheale Meats, Essex, UK) within 4 hours of animal slaughter in antibiotic supplemented HBSS. Aortas were inspected for damage before use. The aorta was first removed from the heart before excision of squares of tissue, approximately 2.5 cm×2.5 cm in area, from regions of interest defined by anatomical landmarks. The ‘outer curvature’ section was taken approximately 0.5 cm proximal to the origin of the bracheocephalic artery and the inner curvature from a region approximately opposite this (Figure 1).

The tissue samples were stripped of excess connective tissue and washed in PBS before they were pinned to wax patches in dishes containing L15 medium without phenol red (Invitrogen, UK) for SICM.

### Scanning Ion conductance microscopy (SICM)

The SICM method has been described previously [17]. Briefly, the SICM probe consists of a glass nanopipette filled with electrolyte. The ion current will decrease the closer the pipette is brought to the sample surface until a predetermined level is reached and a feedback control stops the pipette from moving further. A plot of pipette position creates an image of the cell surface. To improve the reliability of the method we recently introduced hopping mode SICM [20].

Monolayers of PAEC grown with or without shear stress were scanned by SICM (Ionscope). Micropipettes for scanning were pulled using a Laser puller (Sutter Instruments, P-2000); they had a diameter of 500 nm and a resistance of 25 MΩ. Topographic images were obtained by scanning an 80 µm×80 µm or 64 µm×64 µm area in hopping mode. Cultured cells and aortic segments were scanned for a maximum of 2 hours.

### Compliance measurements

**Figure 2:**
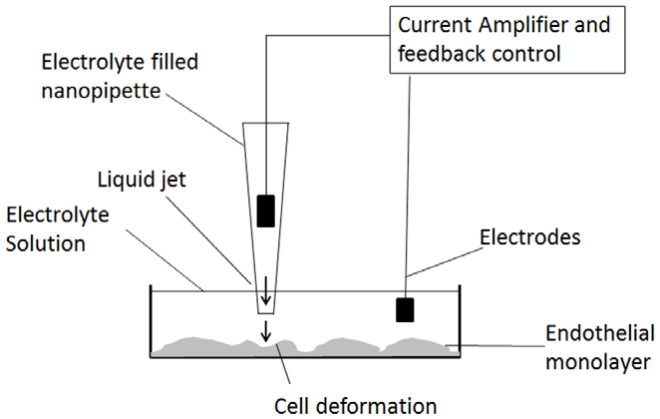
Schematic depiction of the SICM setup in a typical cell compliance experiment.

Using the principle described before [17], SICM was used to determine mechanical properties of cells by subjecting them to locally produced dynamic pressure (see figure 2).

To calculate the compliance of single cells as result of local dynamic pressure, the resistance of the pipette (R), the induced pressure and the resulting displacement of the cell surface were determined. Calculations were performed using the following mathematical formula:
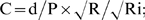
where C = compliance d = displacement of the membrane (µm); P = pressure (kPa); R = actual pipette resistance (Ω); Ri = ideal pipette resistance (Ω).

The displacement measurement, d, was obtained at maximal applied P, which was usually in the range 30–40 kPa. Compliance measurements were calculated only where there was demonstrable recovery of the cell membrane to its original height.

### Calculation of index of elongation and data inclusion

**Figure 3:**
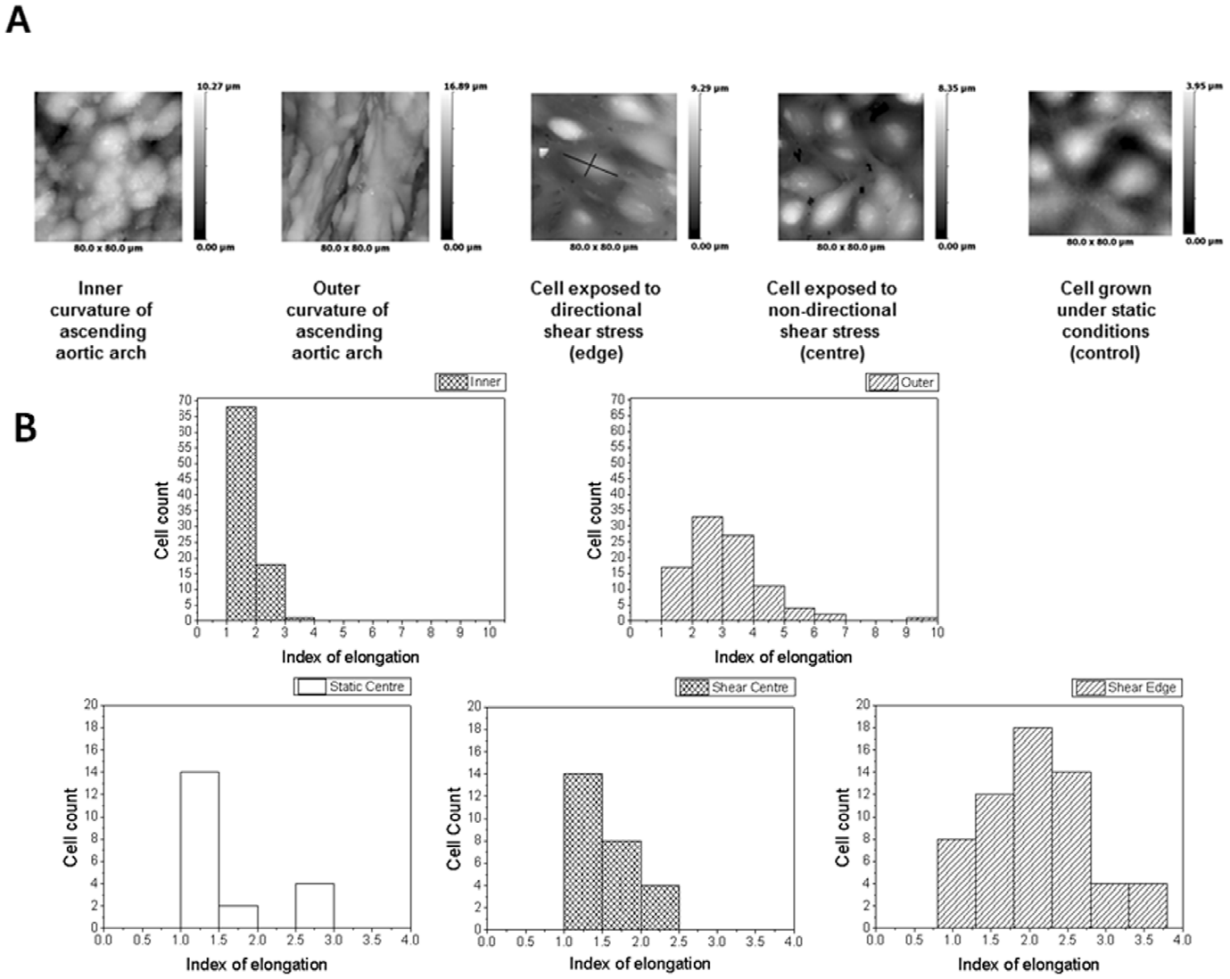
Representative SICM images from different parts of aorta and endothelial cells after shear stress. (A) Representative topographical SICM images of 80×80 µm regions of the inner and outer curvature regions of the ascending pig aorta and of PAEC cultured under static (control) or sheared conditions. The greyscale represents the height of the cells. Edge = a region near the edge of the well thought to experience directional pulsatile shear and centre = a region near the centre of the well thought to experience non-directional steady shear (B) Distribution of IE under all conditions studied. (Upper graphs- inner and outer parts of aorta; Bottom graphs-cell culture-static centre, shear centre and shear edge).

Measurements of elongation were obtained using ScanIC image (Ionscope, UK). Only complete cells with clear borders were included in the analysis. Images were excluded where the endothelial cell surface appeared damaged in any way. Length measurements were taken at the longest point of the cell and width values from the broadest part of the cell. Length was divided by width to obtain the index of elongation (Figure 3 A, third image).

## Results

SICM images revealed that PAEC grown under static conditions and in the centre of a culture well under shear had polygonal morphology whereas PAEC from the edge of the sheared well had an elongated and aligned appearance. Endothelial cells lining the outer curvature of the ascending porcine aorta were also elongated and aligned with each other, whilst cells from the inner curvature were more rounded and had no tendency for alignment (Figure 3A).

Cell morphology was quantified using the Index of Elongation (IE), a calculated length-to-width ratio generated by ScanIC Image. Cells at the sheared well edges had an average IE of 2.02, exceeding the average IE of 1.4 for cells grown in the centres of the same wells. Cells of the outer curvature of the pig aorta had an average IE of 2.61, exceeding that of PAEC grown under all conditions (Figure 3). The cells of the inner curvature of the aorta had an average ratio of 1.7. There was increased variability in alignment in cells from the native aorta as compared to cultured cells as might be expected based on differences between vessel geometries. There was a tendency for PAEC from the outer curvature to have a greater IE than PAEC from the inner curvature, many of which appeared only partially elongated or round (Figure 3B).

**Figure 4:**
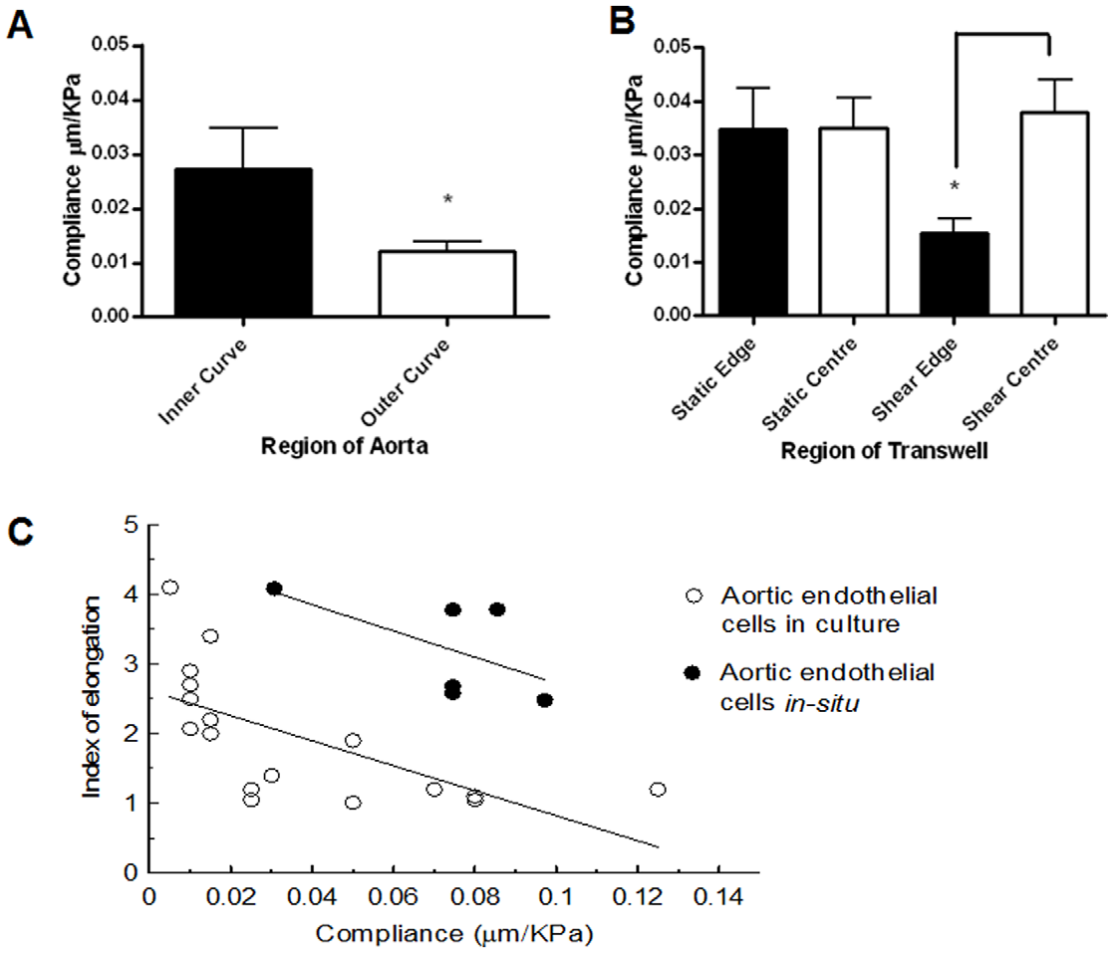
Compliance measurements from different parts of aorta and endothelial cells after shear stress. (A); (B) Compliance of cells of the inner and outer curvatures of the ascending aorta and of cultured PAEC. Data were analysed by T-test. P≤0.05 was deemed significant and is indicated by *. (C) Relation between shape and compliance of cells in the aorta (linear correlation coefficient R = −0.56) and PAEC (linear correlation coefficient R = −0.66).

The SICM probe is a nanopipette that can produce a jet of buffer, and this allowed measurement of cell compliance by local application of a dynamic pressure (Figure 2). The compliance of cells in the outer curvature of the ascending aorta (0.012±0.002 µm/kPa) was close to the compliance of PAEC cultured at the sheared well edge (0.016±0.003 µm/kPa). The compliance of cells in the inner curvature was higher (0.027±0.008 µm/kPa) and approached that of PAEC cultured in the shear well centre (0.038±0.006 µm/kPa). The compliance of cells from all regions of the well cultured under static conditions (0.035±0.006 µm/kPa) was nearly identical to those of PAEC cultured in the shear well centre (Figure 4A).

Figure 4B shows the relationship between index of elongation and compliance for endothelial cells from the in vitro shear stress model and the native aorta. For the range of compliances occupied by cells of both types, there was a remarkable agreement in the slope of the regression lines. Some compliance values measured from the least elongated cells in vitro substantially exceeded any compliances measured in the intact aorta; a large spread was seen in compliance values for these cells.

## Discussion

Scanning ion conductance microscopy (SICM) was invented by Paul Hansma in the 1980s [21] and was later adopted to image and analyse the surface topography of live cells [20], [22], [23]. This was possible because SICM uses a nanopipette as a scanning probe which can be utilised to image cell surface structures with nanometre resolution without touching the cell [23]. Our laboratory further developed this system, introducing a plethora of associated methods [24], [25], [26]. Amongst other methods we developed SICM for measuring cell shape and volume. Recently, we have improved on the reliability and accuracy of SICM by introducing hopping probe ion conductance microscopy (HPICM) [20]. This allows us to obtain nanoscale resolution in highly convoluted live cell samples, such as whole tissue preparations e.g. the organ of Corti, without compromising the scan speed.

Endothelial cell elongation [2] and compliance [13] have both previously been shown to depend on applied shear stress in vitro. Here we used a recently developed cell culture model in conjunction with Scanning Ion Conductance Microscopy to measure elongation and compliance of individual cells exposed to pulsatile, oriented shear or non-pulsatile, non-oriented shear. We found a strong relation between the two cell properties with elongated cells showing reduced compliance. This in in agreement with previous studies carried out using AFM on cells that had experienced an acute application of shear stress [27].

SICM also enabled us to make measurements of endothelial cell compliance from different regions of the aorta. We chose the inner and outer curvature of the ascending aorta; in mice these regions are atheroprone and atheroprotected, respectively [3], and experience different patterns of shear [28], although these properties may not hold for larger species. Differences between the regions of the aorta were similar to those found between the different culture conditions, and there was a similar relation between compliance and elongation. This new application of SICM presents exciting opportunities for future study.
