# Correction: Shape and Compliance of Endothelial Cells after Shear Stress In Vitro or from Different Aortic Regions: Scanning Ion Conductance Microscopy Study

**DOI:** 10.1371/annotation/3356c253-acd6-422b-8bb0-26359b6b0355

**Published:** 2012-05-24

**Authors:** Claire M. F. Potter, Sophie Schobesberger, Martina H. Lundberg, Peter D. Weinberg, Jane A. Mitchell, Julia Gorelik

Information was missing from the Funding section. It should read: This research was supported by the British Heart Foundation through the BHF Centre of Research Excellence at Imperial College London(BHF RE/08/002), BHF Grant (NH/10/3/28574 to JG and JM), Wellcome Trust Grant (085255/Z/08/Z to JM). The funders had no role in study design, data collection and analysis, decision to publish, or preparation of the manuscript.

